# Compressive Strength of Corrugated Paperboard Packages with Low and High Cutout Rates: Numerical Modelling and Experimental Validation

**DOI:** 10.3390/ma16062360

**Published:** 2023-03-15

**Authors:** Lajos Fehér, Damian Mrówczyński, Renáta Pidl, Péter Böröcz

**Affiliations:** 1 Department of Applied Mechanics, Széchenyi István University, Egyetem tér 1, 9026 Győr, Hungary; 2 Research and Development Department, Femat Sp. z o.o., Wagrowska 2, 61-369 Poznań, Poland; 3 Department of Logistics and Forwarding, Széchenyi István University, Egyetem tér 1, 9026 Győr, Hungary

**Keywords:** paperboard packaging, finite element method, box compression test, numerical model, cutout, compression force

## Abstract

The finite element method is a widely used numerical method to analyze structures in virtual space. This method can be used in the packaging industry to determine the mechanical properties of corrugated boxes. This study aims to create and validate a numerical model to predict the compression force of corrugated cardboard boxes by considering the influence of different cutout configurations of sidewalls. The types of investigated boxes are the following: the width and height of the boxes are 300 mm in each case and the length dimension of the boxes varied from 200 mm to 600 mm with a 100 mm increment. The cutout rates were 0%, 4%, 16%, 36%, and 64% with respect to the total surface area of sidewalls of the boxes. For the finite element analysis, a homogenized linear elastic orthotropic material model with Hill plasticity was used. The results of linear regressions show very good estimations to the numerical and experimental box compression test (BCT) values in each tested box group. Therefore, the numerical model can give a good prediction for the BCT force values from 0% cutout to 64% cutout rates. The accuracy of the numerical model decreases a little when the cutout rates are high. Based on the results, this paper presents a numerical model that can be used in the packaging design to estimate the compression strength of corrugated cardboard boxes.

## 1. Introduction

In logistics, the packaging of products is essential [1,2,3,4,5]. The basic function of packaging is to protect packaged products against the effects occurring in logistics. The typical loading conditions in logistics during transport, storage, and loading processes can be divided into two groups: mechanical and climatic loads [6,7,8]. External loads can cause damage to packaging, which can interrupt logistics chains [9,10,11]. Much of the packaging used in logistics is made of corrugated board and can be closed boxes, open boxes on the top, or even sidewall-less boxes. The main advantages of corrugated packaging are its economy, reliable protection of products, relatively low specific weight, low packaging costs, and the recyclability and biodegradability of the paper [12,13]. Corrugated board is always made up of odd layers. The number of layers in practice is 3, 5, or 7. The corrugated layers can be A, B, C as normal corrugated; and D, E, F as micro-corrugated according to the corrugation height [14]. The flat layers are bonded to the corrugated layer with a water-soluble adhesive. Corrugated product manufacturers sell their products in sheet form. From these flat sheets, the packaging manufacturers cut out the expanded form of the packaging material. These cutout flat sheets are delivered to the users. To make the final packaging, the producers fold them in the appropriate way to produce the finished box and fix the sleeves and the top and bottom sheets. As can be seen from the foregoing, the corrugated board packaging is delivered to the place of use in a flat condition, so that it is transported with good volume utilization of the vehicles.

For the production of corrugated sheets, there are several quality grades of both flat and corrugated sheets, basically classified by their fiber content:

Flat sheets can be: kraftliner, which contains only primary pulp fibers; bodyliner (duplex), which contains primary fibers in one part and recycled fibers in the other part; and srenc, which are made by processing mixed waste paper. The papers forming the corrugated layer can be divided into two categories: fluting (semi-chemical), which contains primary fibers and is made suitable for retaining the fluting by special chemical treatment; wellenstoff, which is virtually identical to the base material for flat layers; and srenc.

The paper material is highly sensitive to moisture and all its strength properties are significantly reduced by high moisture content. High fiber content materials (kraftliner, fluting) are less sensitive to moisture than paper materials containing recycled fibers. For this reason, when corrugated board packaging is tested, the type of paper used should always be specified for each layer.

For both papers and corrugated sheets made from them, the direction of manufacture should be interpreted. In the paper industry, the direction of manufacture is abbreviated as MD, whereas the direction perpendicular to the manufacture is abbreviated as CD. The mechanical properties (tensile strength, bending stiffness, compressive strength) of the paper in the production direction are significantly higher than those of the CD. Similarly, the thickness direction (ZD) that can be interpreted for corrugated sheets is important because it determines the second moment of area of the corrugated sheet under compressive loading [15,16,17].

In box manufacturing, FEFCO designates each box type by a numerical code, the simplest and most commonly used box variant being the so-called slotted box, designated by the code FEFCO0201 [14].

In logistics, corrugated boxes are stacked for both storage and transport. The determination of the stacking load capacity for corrugated boxes is still a problem for designers [18].

The best-known empirical formula for sizing for stack loading is the McKee equation, which attempts to determine the compressive strength of a corrugated box based on the Edge Crush Test (ECT) of the corrugated sheet [19]. The ECT of a corrugated board can be estimated by artificial intelligence [20]. Several authors have modified the original equation, and Kellicut and Landt attempted to use the ring crush test (RCT) value [21]. Beldie and co-workers developed a mechanical model for corrugated cardboard boxes subjected to static compressive loading [8]. The authors modelled the corrugated cardboard box as an orthotropic, linearly elastic-plastic laminate. Nowadays, FEM (finite element method) techniques are continuously improving, and several software tools have become available for the strength analysis of statically indeterminate structures. There are several studies in the literature that investigate the numerical analysis of the transverse shear stiffness of corrugated paper sheets [22,23,24,25,26]. The applicable model can, in principle, be simplified by homogenizing the material [27,28,29,30,31]. The corrugated paper sheet is assumed to be a composite material, neglecting that it is made up of multiple different layers. The method of homogenization was demonstrated by Hohe for sandwich panels by basing the approach on strain energy [32]. To investigate the effect of wrinkling on the local strength of corrugated paper sheets, a comparison of laboratory experimental and FEM results was performed by Thakkar et al. [33]. Beex and Peerlings [34] also performed similar experiments. Leminen et al. performed experimental, as well as numerical, studies on the effect of compression creasing on the mechanical properties of corrugated sheet [35].

In practice, it is often necessary to make holes and cutouts of various sizes in the sidewalls of the box for various purposes. These can have several purposes:

- Hand holes for carrying [18];
- Ventilation openings [36];
- Products requiring cold storage;
- Window-like cutouts for reading product identifiers or codes;
- Window-like cutouts to reduce the amount of corrugated board used.

The effect of sidewall cutouts in reducing the compressive strength of the box has been investigated by several authors, but good estimation for BCT value on various sizes and locations of the cutouts has not yet been published. Experiments and modelling have generally been carried out on specific box types used in practice by the authors, and very often, conflicting data have been obtained [7,36].

It would be advisable to measure BCT (box compression test) values for each variant under laboratory conditions in a methodical way, by gradually reducing the surface area of the sidewalls, and to develop a FEM parameterization based on this, which represents the measured BCT values with a good approximation. This procedure could also help designers to place a cut of any shape in the actual cut location, and the model could be used to numerically determine the reduction in BCT value.

## 2. Materials and Methods

### 2.1. Samples

The box samples were made from single-wall B-flute corrugated cardboard material. The material properties of the tested corrugated cardboard are shown in Table 1. The corrugated cardboard contained the following components:

- Outer liner: 210 GD2 (weight 210 $g/m^{2}$, coated white lined chipboard with grey back, quality class 2);
- Fluting medium: 120 HC (weight 120 $g/m^{2}$, high compression Wellenstoff);
- Inner liner: 130 TL 3 (weight 130 $g/m^{2}$, Testliner, quality class 3).

**Figure 1:**
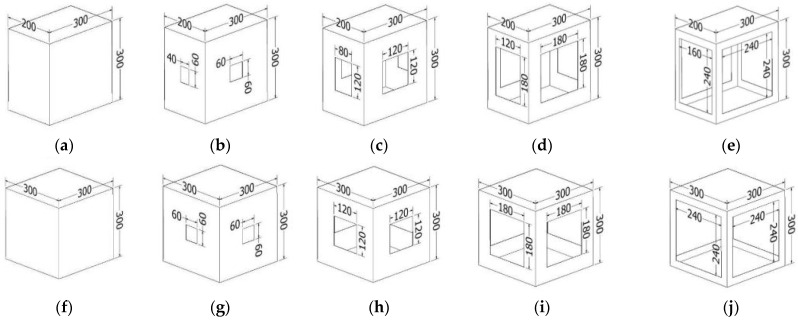

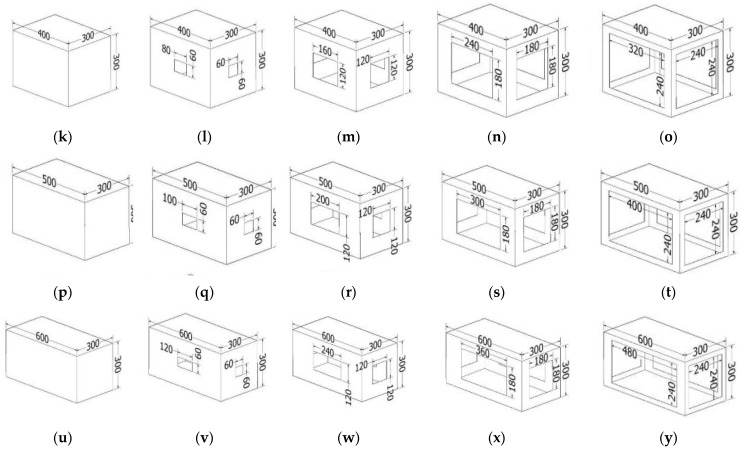
Cases of tested samples with different cutouts. (**a**–**y**) Twenty-five tested samples.

Figure 1 shows the tested box samples. Five different box lengths with 5 different cutout rates were tested; therefore, 25 types of samples were analyzed for this study. The widths and heights of the samples were the same, 300 mm in each case. The length dimension of the boxes varied from 200 mm to 600 mm with a 100 mm increment. The cutout rates were 0%, 4%, 16%, 36%, and 64% with respect to the total surface area of the sidewalls of the boxes. These cutouts were positioned in the middle of the sidewalls of the boxes along all four sides, as shown in Figure 1.

**Figure 2:**
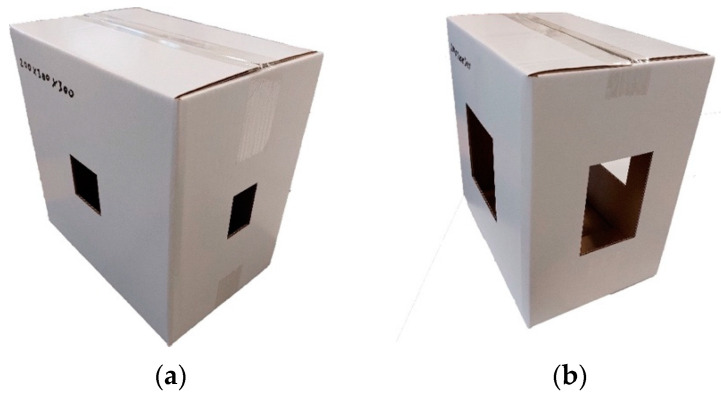

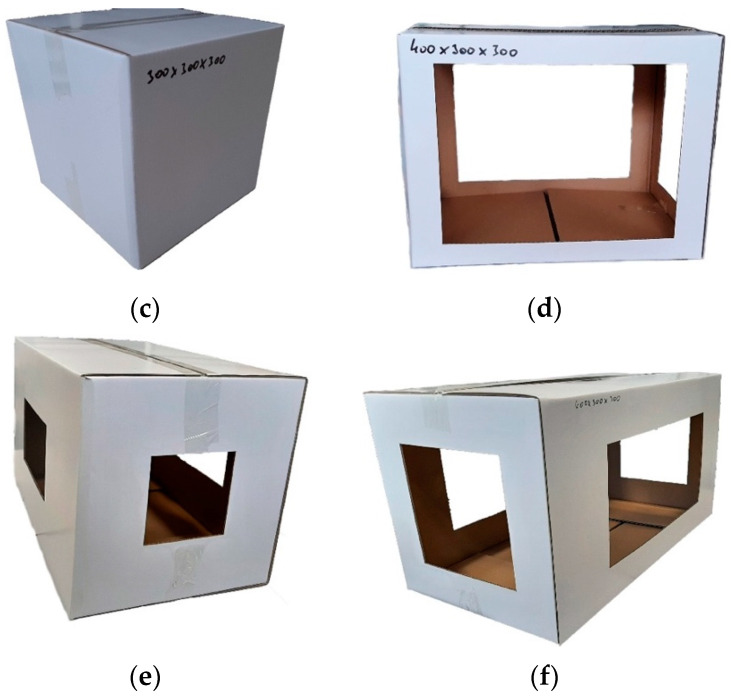
Examples of tested box samples: (**a**) box 200 × 300 × 300 mm with 4% cutout, (**b**) box 200 × 300 × 300 mm with 16% cutout, (**c**) box 300 × 300 × 300 mm with 0% cutout, (**d**) box 400 × 300 × 300 mm with 64% cutout, (**e**) box 500 × 300 × 300 mm with 16% cutout, (**f**) box 600 × 300 × 300 mm with 36% cutout.

Figure 2 shows a few examples of the tested box samples. In Figure 2, each size and cutout group are presented with one or two examples. The exact sizes of the tested box samples and the sizes of the cutouts are shown in Figure 1.

### 2.2. Experimental Setup

**Figure 3:**
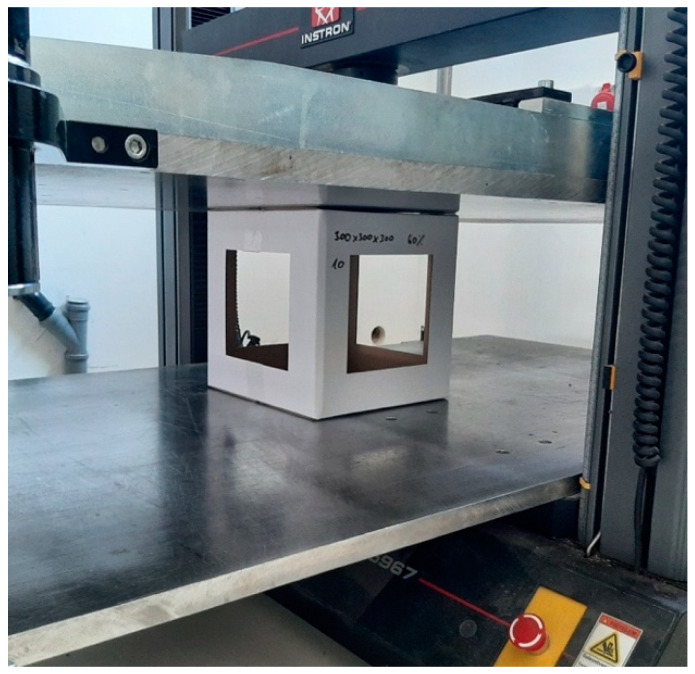
BCT measurement setup.

The experiment consisted of a BCT test (box compression test) to measure the strength behavior of different boxes. The BCT setup is shown in Figure 3. Before the test, the boxes were preconditioned at 30 °C ± 1 °C and 20–30% RH (relative humidity) for 24 h and then conditioned at 23 °C ± 1 °C and 50 ± 2% RH for 24 h in a climate-testing chamber in accordance with the ASTM D4332 standard. Right after the conditioning process, the BCT measurement was performed according to the ASTM D642 standard. The testing speed was 12.7 mm/min ± 2.5 mm/min until failure of the box occurred. The recorded data were the compression force and the deformation, continuously during the measurement. For statistical evaluation, 10 samples were tested for each box design.

### 2.3. Numerical Model of Cardboard Boxes with Different Cutouts

**Figure 4:**
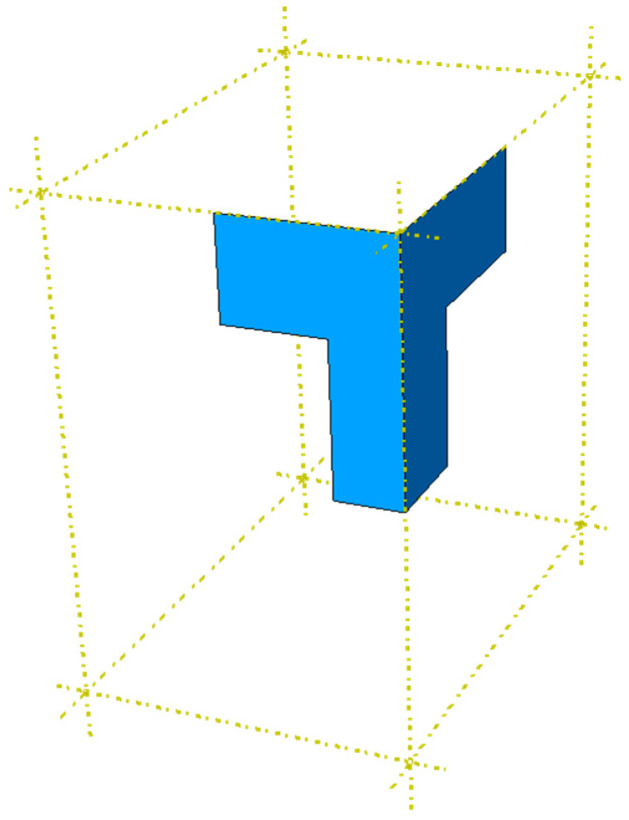
Scheme of the 1/8 parts of the packaging.

Numerical calculations were performed in Abaqus Unified FEA software [37]. Twenty-five different packaging models with the dimensions shown in Figure 1 were built to compute their compressive strength. To speed up and simplify the analysis, only 1/8 of the box was modeled. The top and bottom of the packaging was also omitted because they do not affect the load-bearing capacity. Figure 4 shows an example model of 1/8 parts of the packaging for a case with a length of 200 mm and cutout rates of 36%.

**Figure 5:**
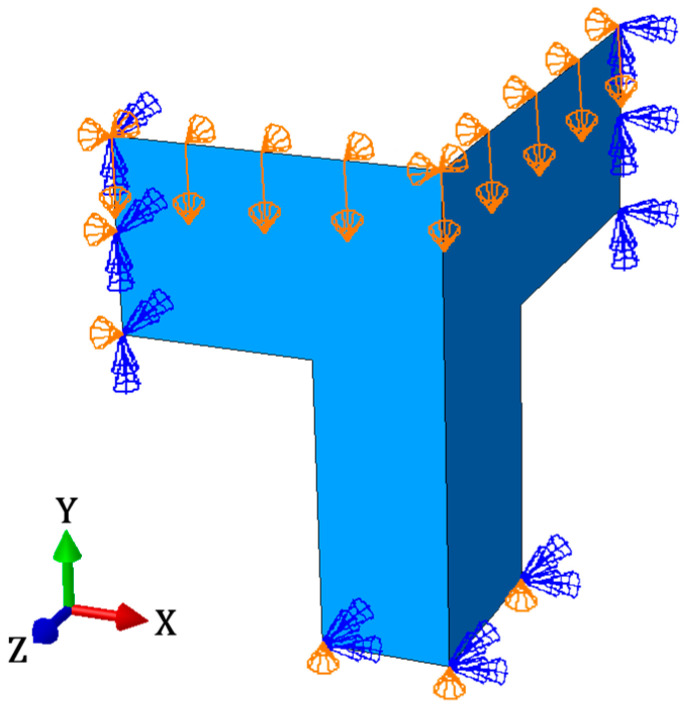
Boundary conditions of the box.

The proper behavior of the box under load was ensured by defining symmetry boundary conditions in the x-direction ($u_{x}=0$, $\varphi_{y}=0$, $\varphi_{z}=0$), y-direction ($u_{y}=0$, $\varphi_{x}=0$, $\varphi_{z}=0$), and z-direction ($u_{z}=0$, $\varphi_{x}=0$, $\varphi_{y}=0$), where $u_{i}$ is the displacement along the i-axis and $\varphi_{i}$ is the rotation angle about i-axis (see Figure 5). The out-of-plane displacement of the top edges was blocked ($u_{x}=0$, $u_{z}=0$), which results from the existence of flaps in the real packaging. A vertical displacement, $u_{y}$, was also applied to the top edges, which simulates the box compression test. The analysis was carried out in two calculation stages. In the first, a buckling analysis was performed to obtain the mode of global imperfections. The previously determined shape of imperfection was applied to the packaging in the second computational step, and it was loaded with a vertical displacement of the top edges in order to obtain the box compressive strength.

In the strength analyses, the linear elastic orthotropic material model was used. Additionally, Hill plasticity was used to differentiate the reference yield strength only in the machine and cross direction [38]. The packaging was made of B-flute cardboard with a grammage of 512 $g/m^{2}$; therefore, the material was marked as B-512. In Table 2, the corrugated board input data to the constitutive model are shown. The mechanical parameters of the material were analytically determined by the BSE System via FEMAT [39] based on the laboratory test data contained in Table 1. Columns 2–7 of Table 2 represent elastic orthotropic material parameters: $E_{1}$ and $E_{2}$ are the moduli of elasticity in the MD and CD, $\nu_{12}$ is the Poisson’s ratio, $G_{12}$ is the in-plane shear modulus, and $G_{13}$ and $G_{23}$ are the transverse shear moduli. Columns 8 and 9 contain plastic material parameters: $\sigma_{0}$ is the initial yield stress and $R_{11}$ is the yield stress ratio in the MD.

For each packaging geometry, buckling analysis and main compression calculations were performed, which gives a total of 50 numerical analyses. The 4-node quadrilaterals shell elements without integration, named S4R, were used in all computations [37]. Different global mesh sizes were assumed for different geometries. For example, for the model shown in Figure 4 and Figure 5, the global mesh size was equal to 5.5 mm, which ultimately resulted in 856 nodes, 783 elements, and 5136 degrees of freedom.

## 3. Results

For each box sample, the maximum compression force values were determined both with measurement and FEM analysis. These experimental and numerical results are shown in Table 3. The results of the numerical tests (Table 3) show that the 400 × 300 × 300 with 0% cutout is the stiffest box, with a compression force of 2731 N. This shows a good match with the experimental data, in which case, the 400 × 300 × 300 box with 0% cutout also has the maximum compression force, 2651 N. The same can be seen for the weakest box because from both tests (numerical, experimental), the 200 × 300 × 300 box with a 64% cutout has the lowest compression force value.

**Figure 6:**
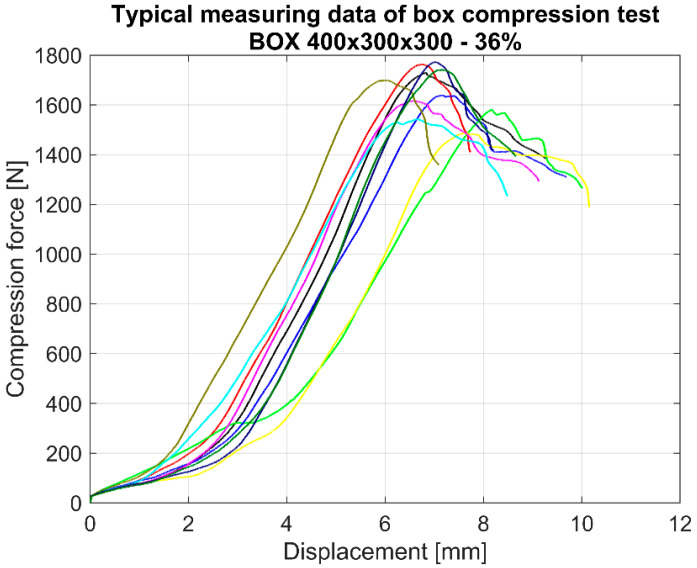
Typical experimental BCT plots for this study.

In Figure 6, typical BCT measuring data can be seen. Figure 6 shows the force displacement recorded data of the 400 × 300 × 300 box with 36% cutout; similar graphs were obtained in each case. There are 10 graphs in Figure 6 due to the number of tested samples being 10 in each box group. The maximum compression force was calculated using the average of the maximum values of the 10 samples.

In Table 4, the percentage differences are shown. If the difference is positive, that means the numerical model overestimates the maximum BCT force, whereas if the value is negative, that shows an underestimation. In only six cases, the percentage difference is greater than 10%. The worst predictions occur in the 64% cutout group due to the absolute average difference being the highest in this group. The 200 × 300 × 300 box with 36% cutout has the highest percentage difference, −20%.

**Figure 7:**
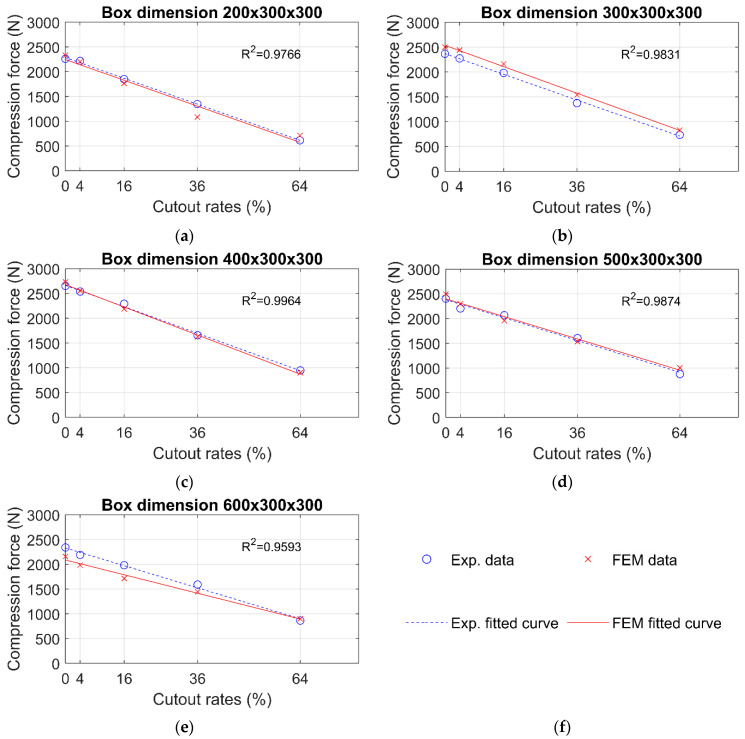
Numerical and experimental maximum compression forces: (**a**) 200 × 300 × 300 mm box, (**b**) 300 × 300 × 300 mm box, (**c**) 400 × 300 × 300 mm box, (**d**) 500 × 300 × 300 mm box, (**e**) 600 × 300 × 300 mm box, (**f**) legend.

In Figure 7, the maximum compression forces are shown, which come from the numerical analysis and the BCT measurements. The dotted blue and continuous red lines represent the linear curves that were fitted in the numerical and the experimental data points. In Figure 7, the $R^{2}$ values (coefficient of determination) are also presented, which come from all data points for the comparison of the numerical and experimental data. These $R^{2}$ values are very high, in the 0.959–0.996 range.

**Figure 8:**
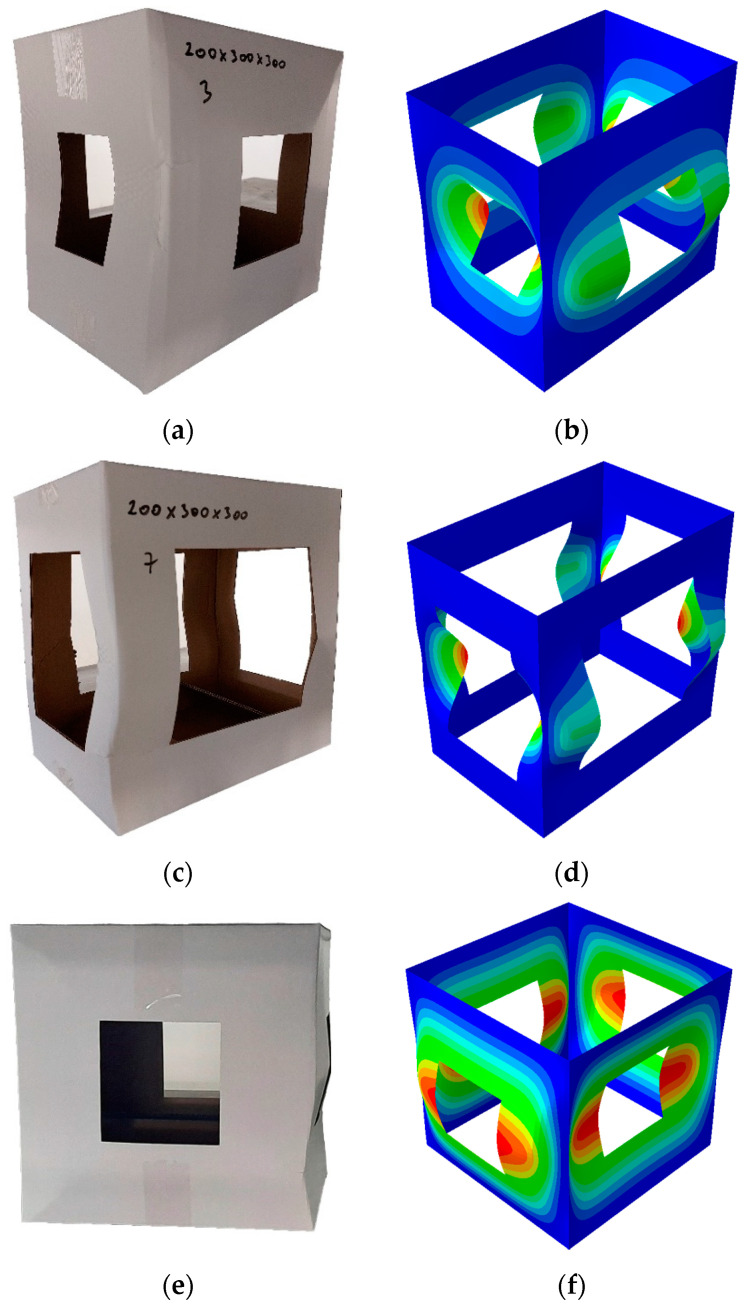
Deformation shapes from experiments and numerical analysis: (**a**) experimental box 200 × 300 × 300 mm with 16% cutout, (**b**) numerical box 200 × 300 × 300 mm with 16% cutout, (**c**) experimental box 200 × 300 × 300 mm with 36% cutout, (**d**) numerical box 200 × 300 × 300 mm with 36% cutout, (**e**) experimental box 300 × 300 × 300 mm with 16% cutout, (**f**) numerical box 300 × 300 × 300 mm with 16% cutout.

A few deformation shape examples are shown in Figure 8. In Figure 8a,c,e, the deformation shapes come from experiments, and on the right side of Figure 8b,d,f, the results come from FEM analysis.

## 4. Discussion

The authors showed the evaluation of the BCT tests for the sample boxes with large cutout areas (Figure 1) in [40,41], and this study is a continuation of those. In this work, the goal was to create a numerical model using the finite element method, which gives good predictions for the BCT values. The finite element method is widely used to model mechanical properties of corrugated boxes [1,8,15,16,18,36,42,43,44,45,46,47,48,49,50,51,52,53,54]. Other authors have conducted similar work [1,2,43,55,56], but in those, the cutout area was not as high as 64%.

Table 3 shows the maximum compression force values from the numerical analysis and from the BCT tests. Comparing these results, it can be seen that the compression force values that come from the numerical analysis are very close to the measured ones. This is even more noticeable in Table 4, which shows the percentage differences between the numerical and experimental compression forces. Overestimation and underestimation of the numerical analysis occur in almost half of the cases, but the differences in most cases are very low. The average absolute percentage difference is 7%. This means the FEM model predicts the reality with a very good accuracy, although the accuracy of the numerical model slightly decreases with higher cutout rates.

In Figure 7, a linear regression is presented. In the previous paper of the authors, it was shown that the linear regression models described the measured data with very high accuracy [40]. This can also be concluded for the data obtained by numerical analysis. Figure 7 shows the $R^{2}$ values (coefficient of determination) that come from all data points. These very high $R^{2}$ values also show a very good fit to the obtained experimental and numerical data.

In some cases, the deformation shapes are very similar in comparison with the numerical model and the experiment. This can be seen in Figure 8a–d. In most of the cases, however, the deformation shapes are different in the same group, since the deformation shapes are highly driven by the imperfections of the boxes. This phenomenon can be seen in Figure 8e–f. These imperfections occur in each case; therefore, the deformation shapes are different if multiple same size boxes with the same cutout rates are tested with BCT. The imperfections could be caused by different things such as the raw material, inappropriate manufacturing or handling, etc.

The results show that the BCT results of the boxes from a low to high cutout rate can be predicted with a high accuracy using this numerical model. The novelty of this paper is to show the ability of FEM analysis to estimate the BCT results of corrugated cardboard boxes with very high cutout rates. In this work, a wide range of the box sizes and the cutout rates were involved, but this range is not comparable with the different type of boxes used in the industry; therefore, all findings apply only to the tested box types. Therefore, a future study should investigate to design and test a box for industry usage with a higher cutout rate.

Moreover, the presented numerical model for this study provides a cost-effective and efficient alternative way in comparison to the traditional experimental testing methods, which can be time-consuming and expensive. From a practical point of view, the presented numerical method is accurate enough for the authority of use. By using numerical simulations, the number of physical prototypes needed for testing can be significantly reduced, leading to cost savings in the design and development of corrugated boxes with cutouts. It also has to be mentioned that the numerical model can be used to perform parametric studies, where the effect of different cutout sizes on the BCT values can be evaluated without the need for additional physical testing.

Overall, the results of this study demonstrate the potential of using finite element method simulations to accurately predict the BCT values of corrugated boxes with larger cutout areas than what has been shown before. The good estimation for the numerical and experimental results, as well as the low average absolute percentage difference, indicate that the developed model is a reliable tool for predicting the performance of corrugated boxes in real-world applications. Furthermore, this study highlights the importance of considering the effect of cutout area on BCT values, as this can significantly impact the strength and durability of corrugated boxes.

## 5. Conclusions

This paper presents a numerical simulation and experimental verification method for the investigation of cutout problems of a single-wall corrugated board box. Although the method of choosing samples for the experimental test follows theoretically located and sized cutouts, the numerical result of analysis shows surprising accuracy in load capacity estimating for a corrugated cardboard box, the structure of which is basically made by viscoelastic material. The accuracy of the model decreases how the cutout rate increases from 0% to 64%. The results give new information for engineers to better understand the strength reduction effect of cutout holes such as carrying or ventilation holes.

## Figures and Tables

**Table 1:** Material properties of corrugated cardboard used in the study.

Properties	Specification	Standard
board thickness	2.8 mm	ISO 3034 (FEFCO no. 3)
grammage	512 $g/m^{2}$	ISO 536:1995
edge crush test (ECT)	5.1 kN/m	ISO 3037 (FEFCO no. 8)
bending stiffness (BS)	4.23 Nm (MD)	ISO 5628:1990
	2.90 Nm (CD)	
bursting strength (BST)	676 kPa	ISO 2759 (FEFCO no. 4)

**Table 2:** Material parameters of the B-flute corrugated board.

Grade	$E_{1}$	$E_{2}$	$\nu_{12}$	$G_{12}$	$G_{13}$	$G_{23}$	$\sigma_{0}$	$R_{11}$
	(MPa)	(MPa)	(-)	(MPa)	(MPa)	(MPa)	(MPa)	(-)
B-512	2149	1474	0.36	3348	3	5	1.83	0.86

**Table 3:** Maximum compression force values (N).

Type	*L*	Cutout Rates (%)				
	(mm)	0	4	16	36	64
experimental	200	2261	2218	1851	1347	615
	300	2367	2275	1981	1373	734
	400	2651	2537	2291	1656	946
	500	2402	2203	2066	1603	877
	600	2339	2189	1980	1591	862
numerical	200	2333	2202	1763	1082	712
	300	2501	2440	2163	1539	828
	400	2731	2556	2185	1625	899
	500	2494	2296	1959	1534	1005
	600	2158	1983	1711	1445	898

**Table 4:** Differences between the numerical and experimental compression force results.

L (mm)	Cutout Rates (%)				
	0	4	16	36	64
	Percentage Difference				
200	3%	−1%	−5%	−20%	16%
300	6%	7%	9%	12%	13%
400	3%	1%	−5%	−2%	−5%
500	4%	4%	−5%	−4%	15%
600	−8%	-9%	−14%	−9%	4%

## Data Availability

The data published in this research are available on request from the first author and corresponding authors.
